# Supplementary material for: Glyco engineered pentameric SARS-CoV-2 IgMs show superior activities compared to IgG1 orthologues
Source: Front Immunol. 2023 Jun 8;14:1147960. doi: 10.3389/fimmu.2023.1147960 (PMC10285447; doi:10.3389/fimmu.2023.1147960)
Supplement: Supplementary file 1 [file DataSheet_1.docx]

Supplementary Material

**Glyco-engineered pentameric SARS-CoV-2 IgMs show superior activities compared to IgG1 orthologues**

Somanath Kallolimath^1^*, Roman Palt^1^*, Esther Föderl-Höbenreich^2^, Lin Sun^1^, Qiang Chen^3^, Florian Pruckner^1,4^, Lukas Eidenberger^1^, Richard Strasser^1^, Kurt Zatloukal^2^ and Herta Steinkellner^1^

* These authors contributed equally

Herta Steinkellner

Department of Applied Genetics and Cell Biology, University of Natural Resources and Life Sciences, Muthgasse 18, 1190 Vienna, Austria

Tel: +43-1-47654-94370,

Fax: 43-1-47654- 94009,

Email: [herta.steinkellner@boku.ac.at](mailto:herta.steinkellner@boku.ac.at)

**Supporting Information:**

**Figure S1:** Peptide sequences of P5C3- and H4-IgG1, -IgM heavy chains, joining chain (JC, P5C3- and H4- κLC kappa light chains. Variable domain is underlined, conserved glycosites are indicated in red**.**

**Figure S2: A:** Schematic presentation of human IgG1, IgM and J-chain, including their serum glycosylation status. Dots represent glycosylation sites (GS); numbering of IgG1 and J-chain refers to GS position; **B:** N-glycan symbols according to Consortium for Functional Glycomics (<http://www.functionalglycomics.org/>).

**Figure S3:** mAbs were evaluated for neutralization potency against SARS-CoV-2 infection measured by plaque reduction assay. Shown values correspond to a representative experiment (each concentration was tested in duplicates).

**Figure S4:** A: Indirect ELISA: antigen-binding activity of P5C3 and H4 mAbs to recombinant RBD, using anti- human IgG-HRP and anti-human IgM-HRP for detection. X-axis: concentration (ng/mL); y-axis: absorbance (AU); B: Ag-binding assay with and without (+Urea and -Urea); EC50 values in (pM: picomoles/L and ng/mL). IgG1 and IgM P: pentamers, respectively.

**Table S1:** Detailed description of site-specific N-glycan composition of different mAbs and JC. Number represent abundance in %; nonglyco refers to % of non-occupied glyco-site. N-glycan symbols according to Consortium for Functional Glycomics (http://www.functionalglycomics.org/).
