# Supplementary figures and images for: Glyco engineered pentameric SARS-CoV-2 IgMs show superior activities compared to IgG1 orthologues
Source: Front Immunol. 2023 Jun 8;14:1147960. doi: 10.3389/fimmu.2023.1147960 (PMC10285447; doi:10.3389/fimmu.2023.1147960)

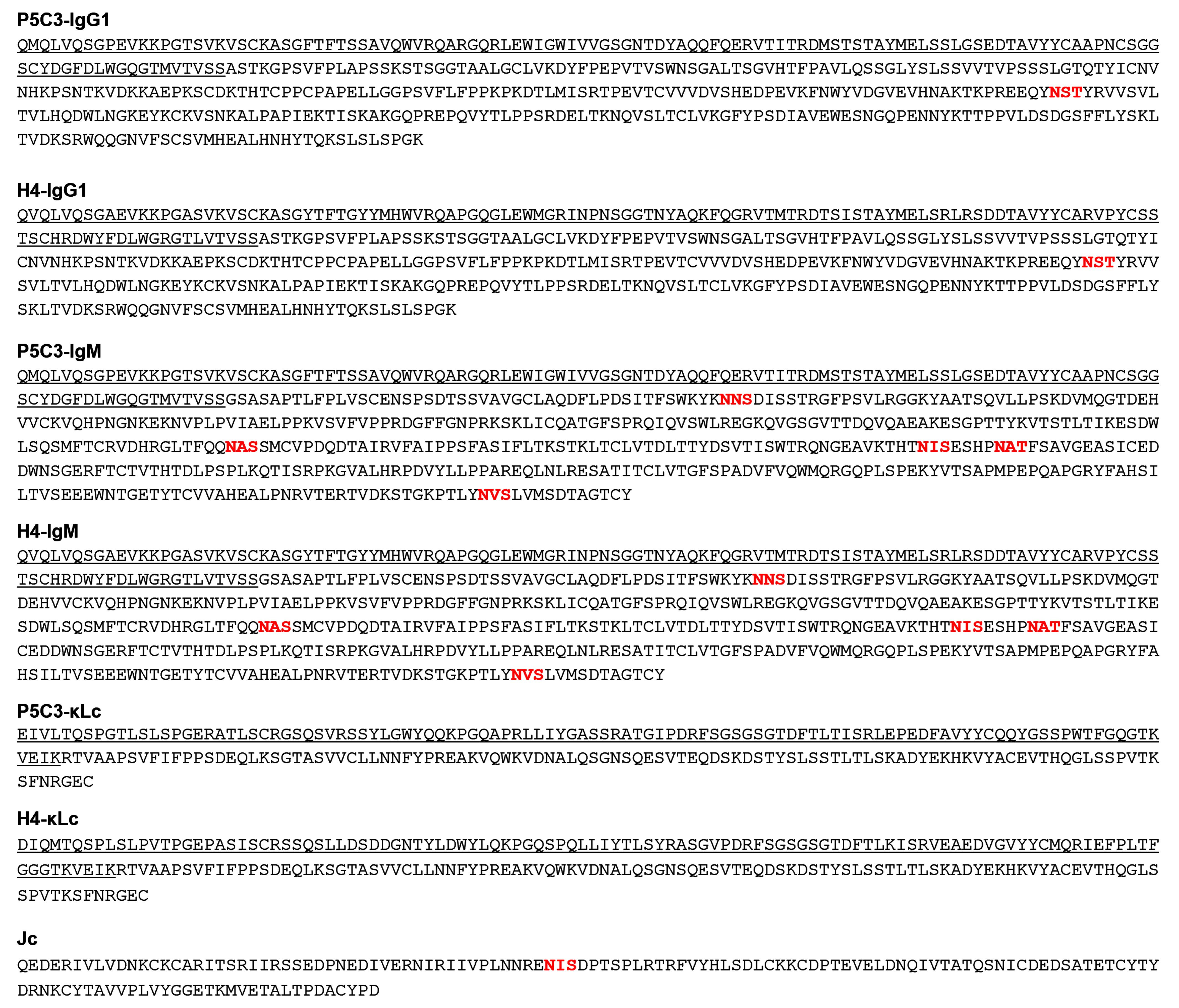

Supplement: Supplementary file 2 [file Image_1.tiff]

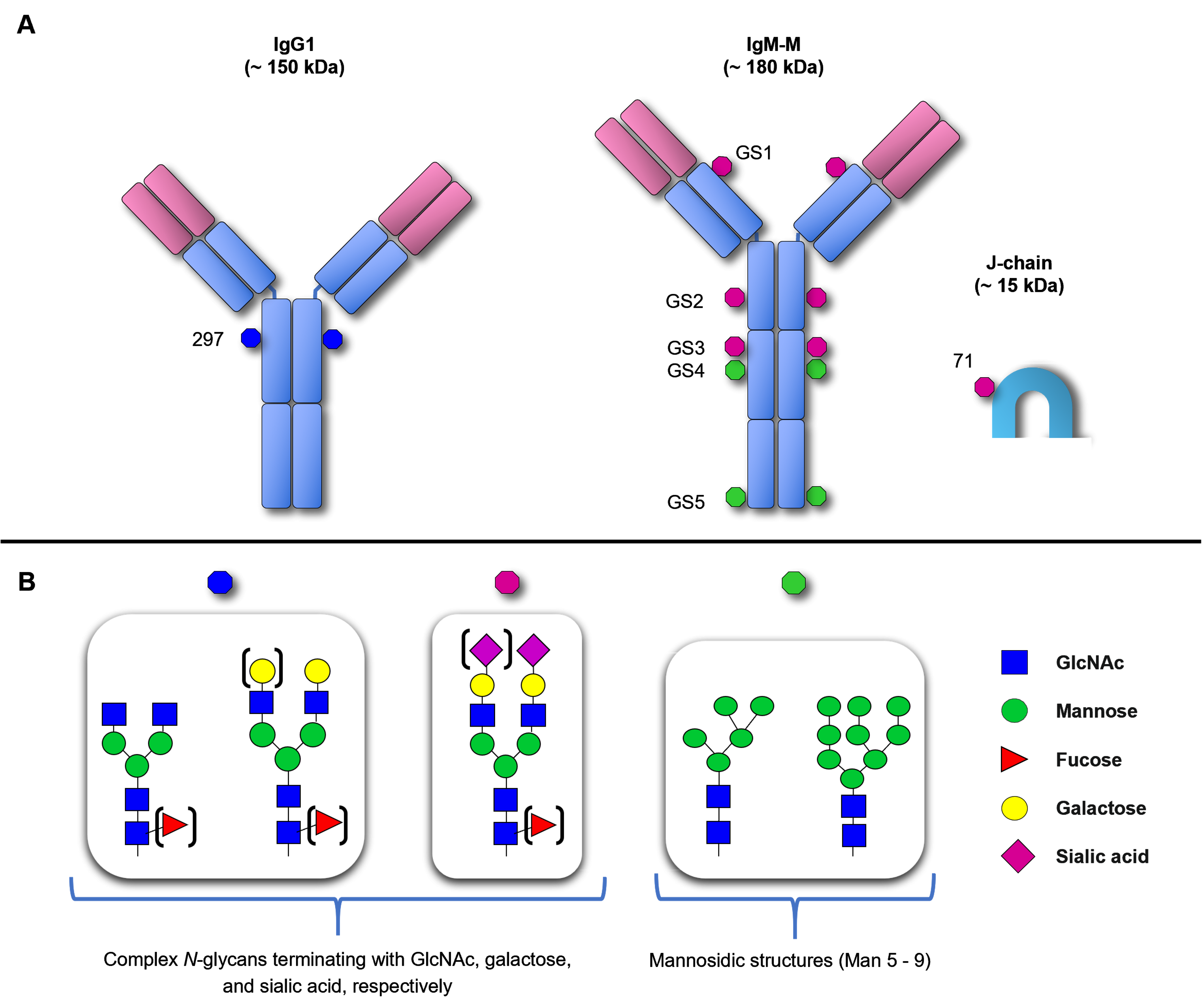

Supplement: Supplementary file 3 [file Image_2.tiff]

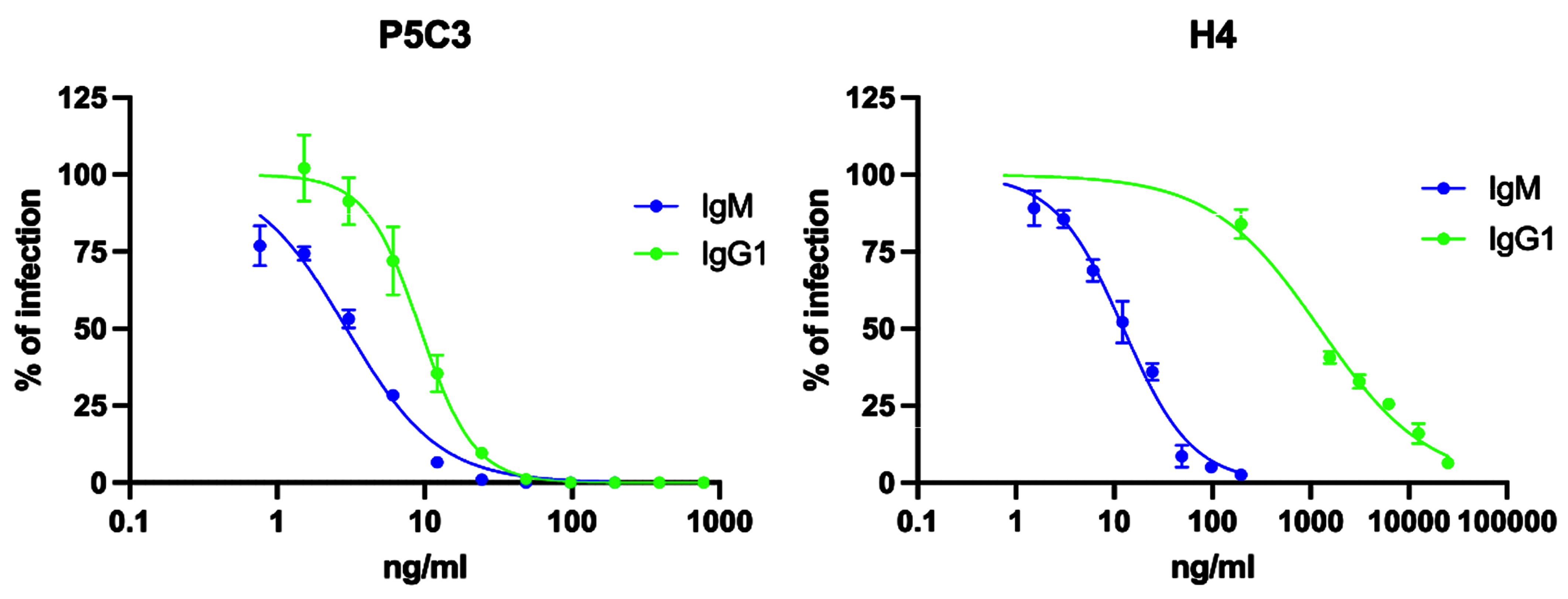

Supplement: Supplementary file 4 [file Image_3.tiff]

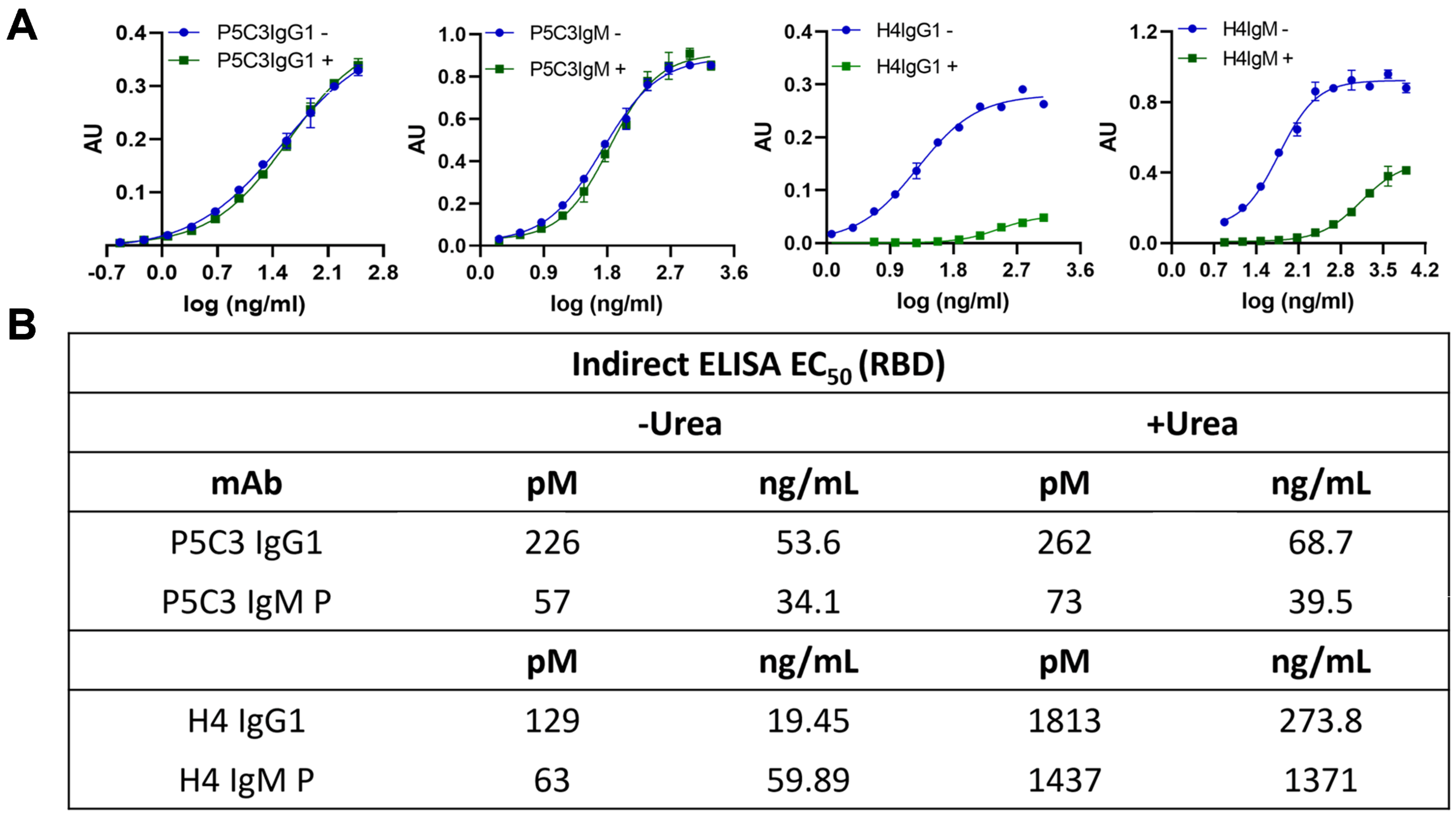

Supplement: Supplementary file 5 [file Image_4.tiff]
