# Supplementary material for: Glyco engineered pentameric SARS-CoV-2 IgMs show superior activities compared to IgG1 orthologues
Source: Front Immunol. 2023 Jun 8;14:1147960. doi: 10.3389/fimmu.2023.1147960 (PMC10285447; doi:10.3389/fimmu.2023.1147960)
Supplement: Supplementary file 6 [file Table_1.docx]

| 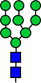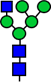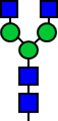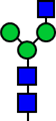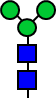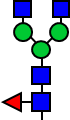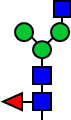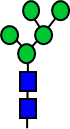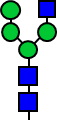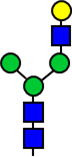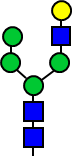 | | | | | | | | | | | | | | | | | | | |  |
| --- | --- | --- | --- | --- | --- | --- | --- | --- | --- | --- | --- | --- | --- | --- | --- | --- | --- | --- | --- | --- |
| ***P5C3-***  ***IgM P*** | **Man 5 - 9** | **GnGn** | **MGn** | | **MM** | | **GnGnF** | | **MGnF** | **Man4Gn/ AM** | | | **Man5Gn/ Man4A** | | **others** | | **Sum** | **non- glyco.** | |  |
| GS1 | 6.8 | 17.7 | 55.2 | | 2.0 | | 8.3 | | 5.8 | - | | | - | | 4.2 | | 100 | 1.0 | |  |
| GS2 | 22.5 | 53.0 | 5.0 | | - | | 14.3 | | 1.2 | - | | | - | | 4.0 | | 100 | 5.1 | |  |
| GS3 | 2.7 | 85.7 | 3.0 | | - | | 7.0 | | - | - | | | - | | 1.6 | | 100 | 43.5 | |  |
| GS4 | 97.9 | - | - | | - | | - | | - | - | | | - | | 2.1 | | 100 | 11.0 | |  |
| GS5 | 97.3 | - | - | | - | | - | | - | - | | | - | | 2.7 | | 100 | 56.2 | |  |
| JC | 22.8 | 19.2 | 21.6 | | 6.8 | | - | | - | 15.4 | | | 14.2 | | 0.0 | | 100 | 0.7 | |  |
|  |  |  |  | |  | |  | |  |  | | |  | |  | |  |  | |  |
| ***P5C3***  ***- IgM M*** | **Man5 - 9** | **GnGn** | **MGn** | | **MM** | | **GnGnF** | | **MGnF** | **Man4Gn/ AM** | | | **Man5Gn/ AGn** | | **others** | | **Sum** | **non- glyco.** | |  |
| GS1 | 33.7 | 13.1 | 39.6 | | 1.7 | | 6.1 | | 3.7 | - | | | - | | 2,2 | | 100 | 1.0 | |  |
| GS2 | 69.7 | 16.8 | 3.0 | | 0.2 | | 5.5 | | - | - | | | - | | 4.9 | | 100 | 4.0 | |  |
| GS3 | 23.0 | 62.7 | 5.4 | | 1.7 | | 6.6 | | - | - | | | - | | 0.6 | | 100 | 59.0 | |  |
| GS4 | 98.5 | - | - | | - | | - | | - | - | | | - | | 1.5 | | 100 | 10.9 | |  |
| GS5 | 97.1 | 1.7 | - | | - | | - | | - | - | | | - | | 1.3 | | 100 | 30.9 | |  |
|  |  |  |  | |  | |  | |  |  | | |  | |  | |  |  | |  |
| ***P5C3***  ***- IgG1*** | **Man5 - 9** | **GnGn** | **MGn** | | **MM** | | **GnGnF** | | **MGnF** | **Man4Gn/ AM** | | | **Man5Gn/ AGn** | | **others** | | **Sum** | **non- glyco.** | |  |
| GS1 | 8.5 | 83.7 | 7.2 | | - | | - | | - | - | | | - | | 0.6 | | 100 | 27.2 | |  |
|  |  |  | |  | |  | |  | | |  |  | |  | |  | |  |  | |
